# Linking alternative reproductive tactics and habitat selection in Northern chamois

**DOI:** 10.1002/ece3.7554

**Published:** 2021-05-01

**Authors:** Luca Corlatti, Antonella Cotza, Luca Nelli

**Affiliations:** ^1^ Chair of Wildlife Ecology and Management University of Freiburg Freiburg Germany; ^2^ Research Unit of Behavioural Ecology Ethology and Wildlife Management University of Siena Siena Italy; ^3^ Institute of Biodiversity, Animal Health and Comparative Medicine University of Glasgow Glasgow UK

**Keywords:** mating behavior, reproduction, resource selection, space use, territoriality, ungulate

## Abstract

In polygynous ungulates, males may achieve fertilization through the use of alternative reproductive tactics (ARTs), discrete phenotypic variations evolved to maximize fitness. ARTs are often associated with different male spatial strategies during the rut, from territoriality to female‐following. Although variation in space use patterns of rutting male ungulates is known to be largely affected by the spatial distribution of females, information on the year‐round habitat selection of alternative reproductive types is scant. Here, we investigate the seasonal variation in habitat choice of a large mammal with ARTs (territoriality and nonterritoriality), the Northern chamois *Rupicapra rupicapra*. Global Positioning System (GPS) data on 28 adult males were collected between February 2010 and December 2013 in the Gran Paradiso National Park (Italy) and used to fit resource selection functions to explore the ART‐specific use of key topographic features, such as elevation, aspect, and slope, and vegetation phenology expressed as NDVI values. Territorial and nonterritorial chamois profoundly differed in their habitat selection not only during the rutting season. Compared to nonterritorial males, territorial males used lower elevations in summer and autumn, preferred southern slopes in spring and summer, and used steeper areas in summer but not in winter. We found no difference in seasonal selection of NDVI values between males adopting ARTs. Our results suggest that territorial males tend to occupy warmer, lower‐food‐quality habitats in late spring and summer, whereas nonterritorial males are free to follow and exploit vegetation phenology and more favorable temperatures. Different patterns of habitat selection may reflect different trade‐offs between the optimization of energy balances throughout the year and the increase of mating opportunities during the rut in males adopting alternative reproductive tactics.

## Graphical abstract

Variation in space use patterns of rutting male ungulates is known to be largely affected by the spatial distribution of females. Information on the year‐round habitat selection of males using alternative reproductive types is scant. In Alpine chamois Rupicapra rupicapra, territorial and nonterritorial males profoundly differed in their year‐round habitat selection patterns, possibly reflecting different trade‐offs between the optimization of energy balances and the increase of mating opportunities.

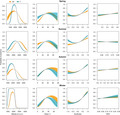

## 1 INTRODUCTION

Habitat use, the way animals use resources for foraging, resting, nesting, denning, or escaping (Krausman, 1999), reflects the need to optimize energy demand, nutrient balance, and individual reproductive success (MacArthur, 1972). Habitat selection occurs when habitat features are used disproportionately to their availability (Hall et al., 1997; Johnson, 1980), and reproductive success and survival may be seen as the main selective pressures that contribute to the evolution of habitat selection mechanisms (Hilden, 1965; Krausman, 1999).

In mammals, habitat use depends on a number of variables. These include biotic factors such as quality, quantity, and dispersion of food resources (Anderson et al., 2005; Nielsen et al., 2010) and abiotic factors such as ambient temperature (Pigeon et al., 2016), snow presence (Dussault et al., 2005; Rivrud et al., 2010), topography (Kie et al., 2005), and presence of shelters (Lucherini et al., 1995). Additionally, individual factors such as sex (Villaret et al., 1997), physiology (e.g., estrus period: Dahle & Swenson, 2003; José & Lovari, 1998), body mass (du Toit & Owen‐Smith, 1989), and presence of offspring (Dahle & Swenson, 2003; Dussault et al., 2005) may also influence habitat use. Habitat selection may be further impacted by intra‐ and interspecific competition (Kjellander et al., 2004) and seasonality (Bjørneraas et al., 2011; Lott, 1991).

In mammals, males and females typically employ different strategies to maximize their fitness (Clutton‐Brock, 1989; Emlen & Oring, 1977). Males, for example, prioritize the maximization of energy gain (Main, 2008) and the increase in mating opportunities (Clutton‐Brock, 1989; Emlen & Oring, 1977), while females prioritize survival and successful rearing of offspring (Clutton‐Brock, 1989). This, in turn, may lead to different strategies of spatial behavior between sexes (Bonenfant et al., 2004) and potentially to sexual segregation, that is, differential space, habitat, or forage use by the two sexes outside the mating season (see Bowyer, 2004 for a review). Hypotheses explaining habitat segregation, especially in ungulates, include differences in reproductive strategies and in risk of predation between sexes (the “reproductive strategy” or “predation‐risk” hypothesis), differences in body size and selection of forage (the “sexual dimorphism‐body size” or “forage‐selection” hypothesis), differences in body size and activity budget (“activity budget” hypothesis), and differences in social preferences (the “social factor” hypothesis) (Bowyer, 2004; Main et al., 1996; Ruckstuhl & Neuhaus, 2000, 2002).

Space use pattern in males, especially during the mating season, is largely affected by the spatial distribution of females during the rut (Ostfeld et al., 1985), which in turn influences male mating system and success (Emlen & Oring, 1977; Maher & Lott, 2000). Habitat choice by males therefore results from an optimal trade‐off between opportunity for survival and opportunity for reproduction, and its understanding may be further complicated by variation within mating systems, that is, by the presence of alternative reproductive tactics (ARTs, alternative ways to obtain fertilization and maximize reproductive success: Taborsky et al., 2008). ARTs are common in polygynous ungulates (Isvaran, 2005) and include, for example, resource‐based territoriality, lekking behavior, tending or coursing behavior (Bowyer et al., 2020; Isvaran, 2005; Wolff, 2008). Reproductive costs and benefits may vary among tactics (Corlatti et al., 2012; Saunders et al., 2005) and different trade‐offs may lead to the exploitation of different reproductive niches (Taborsky et al., 2008). In some species, ARTs can thus be associated with trophic niche divergence, different foraging strategies, or spatial segregation (e.g., Chao et al., 2013; Corlatti, Bassano, et al., 2013; Lattanzio & Miles, 2016). Habitat selection, for example, may be influenced by mating tactic in roe deer *Capreolus capreolus* (Mysterud, 1999). Different space use associated with different mating behavior during the mating season has been reported in different taxa (e.g., insects: Opaev & Panov, 2016; fish: Afonso et al., 2008; amphibians: Forester & Thompson, 1998; reptiles: Shine et al., 2005; birds: Andersson, 1980 and Nelli et al., 2016; mammals: Sandell, 1989; among ungulates: Lovari et al., 2008 for female roe deer, Corlatti et al., 2020 for Northern chamois *Rupicapra rupicapra*). To our knowledge, however, no information is available on year‐round space use variation in individuals adopting ARTs.

In male Northern chamois, two ARTs occur: territoriality and nonterritoriality (Corlatti et al., 2012; Krämer, 1969). During the November rut, territorial males actively defend an exclusive area at relatively low elevations (Corlatti et al., 2012; Krämer, 1969; von Hardenberg et al., 2000), while nonterritorial males display following behavior and territory intrusions (Corlatti et al., 2012; Krämer, 1969; von Hardenberg et al., 2000). ARTs profoundly differ in several ecological and physiological aspects during the rut (Corlatti & Bassano, 2014; Corlatti, Bassano, et al., 2013; Corlatti et al., 2012, 2019). In the rest of the year, on the other hand, such differences are milder (*cf*. Corlatti et al., 2019). Information on space use in territorial and nonterritorial male chamois is limited: Lovari et al. (2006) investigated alternative strategies of space use, supporting the occurrence of resident and migrant males, which may partly associate with ARTs. The hypothesis that in ruts with early abundant snowfalls, females would move to low elevations, thereby favoring mating opportunities in territorial males, while in years with scarce or delayed snowfalls, females would stay at high elevations, thus favoring mating opportunities in nonterritorial males (Lovari et al., 2006) was recently supported by GPS data (Corlatti et al., 2020). Although seasonal habitat selection was studied in Northern chamois (Lovari et al., 2006; Nesti et al., 2010; Unterthiner et al., 2012), no information on ART‐specific spatial behavior outside the rut is available.

The onset of chamois territorial behavior is in late spring (von Hardenberg et al., 2000) and previous studies suggested that territorial males tend to occupy lower elevations than nonterritorial males throughout the year (Corlatti, Bassano, et al., 2013). In this study, we evaluate whether there are differences in seasonal patterns of habitat selection between ARTs, in terms of altitude, slope, and aspect, which are important determinants of temperature (Apaydin et al., 2011), precipitation (Basist et al., 1994), and snow accumulation (Jost et al., 2007), thus indirectly influencing foraging opportunities in herbivores. We also investigated differences in seasonal selection of vegetation productivity, expressed as Normalized Difference Vegetation Index (NDVI) values (Rouse et al., 1974; Tucker et al., 1985). Previous studies correlated NDVI values with grass biomass (Schino et al., 2003) and fecal crude protein content or nitrogen fecal content, considered indicators of vegetation quality (Hamel et al., 2009). We anticipated that territorial males should positively select lower elevations, steeper and south‐facing slopes since springtime, when they start defending their territories, as those areas will be snow‐free during the rut, therefore more attractive to females. Nonterritorial males, on the other hand, would move to higher elevations in spring–summer, following the vegetation green‐up. Thus, NDVI values of areas occupied by territorial males, especially in spring and summer, should be lower than those of areas used by nonterritorial males. At the end of the mating season, when foraging opportunities are constrained by snow cover, we expect no differences in habitat selection between male tactics.

## 2 MATERIALS AND METHODS

### 2.1 Study area and population

**FIGURE 1:**
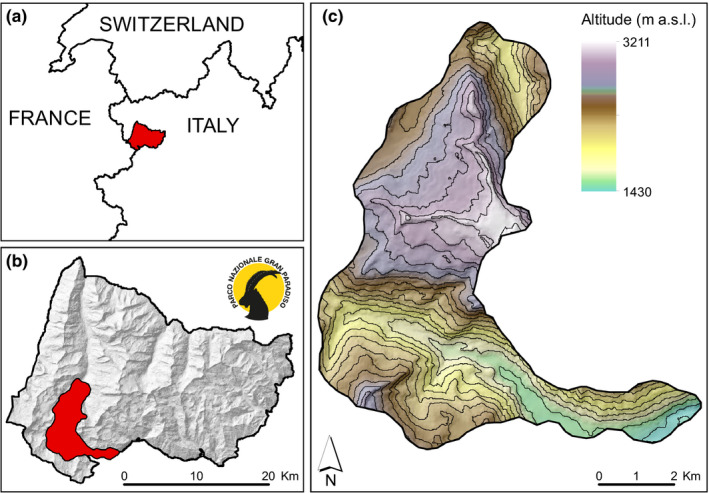
(a) Location of the Gran Paradiso National Park (red area, Western Italian Alps); (b) location of the study site (red area) in the southwestern part of the Gran Paradiso National Park; (c) topographic features of the study area

The study was conducted in the upper part of the Orco Valley and of the Valsavarenche Valley, within the Gran Paradiso National Park (hereafter GPNP, Western Italian Alps, 45°26′30″N, 7°08′30″E) (Figure 1). The area extends over 66.3 km^2^, with an average altitude of 2,346 m a.s.l. (min 1,344 m, max 3,393 m). During the study period 2010–2013, the area was characterized by daily mean precipitation between 2.7 mm in winter and 4.5 mm in autumn and by daily minimum average temperature between −7.8°C in winter and +7.3°C in summer (own data; for the definition of season, see paragraph Statistical analysis below). Within the study site, vegetation on south‐facing slopes is quite homogeneous, consisting of graminoids, forbs, and shrubs. At the lowest elevations of the south‐facing slopes, there are mixed woods of larch *Larix decidua*; however, most of the area is characterized by xero‐thermophilous grasslands, dominated by meadows of colored fescue *Festuca varia*, associated with sedges *Carex* sp. and rush *Juncus* sp. The north‐facing slopes are characterized by alpine and subalpine meadows of Alpine sedge *Carex curvula* at higher elevations and woods of larch and patches of alder shrubs *Alnus viridis* at lower elevations. The male chamois population in the GPNP has been protected since 1922, and it shows summer densities of approximately 10 ind./km^2^ (Corlatti, Fattorini, et al., 2015).

Between 2010 and 2012, 28 adult (3.5–12 years old) male chamois were ground‐darted by the Park personnel with a combination of xylazine and ketamine. Captures were always performed with the presence of a veterinarian, in accordance with the Italian law. During captures, each animal was equipped with an individually recognizable Global Positioning System (GPS) collar (Vectronic Aerospace GmbH). Animals were classified into “territorial” (“T,” *n* = 12) or “nonterritorial” (“NT,” *n* = 16). The distinction between male types was based on the cluster analysis of behavioral patterns and space use during the mating season, assuming that territorial males would have higher site fidelity and win more intrasexual interactions than nonterritorial males. Specifically, for each individual, the home range (90% fixed kernel) was calculated using high‐quality GPS locations, that is, with at least four satellites and dilution of precision values lower than 10 (Lewis et al., 2007), and individual tracks were kernel‐smoothed with the plug‐in bandwidth selector (“hpi”) of Wand and Jones (1994). The proportion of intrasexual interactions won was calculated using behavioral data recorded throughout the mating season, during hourly sessions of observations ad libitum (Altmann, 1974) on each individual. A male was considered as winner if the opponent was chased away or displayed submissive behaviors. These two parameters were combined in a matrix, and multivariate hierarchical clustering (Everitt et al., 2011) using the Mahalanobis distance (Mahalanobis, 1936) was conducted. Males were classified as territorial if they had small home ranges and high values of intrasexual interactions won.

Details of the statistical procedure for the distinction between ARTs are reported in Corlatti et al. (2012), while full data and codes are available from Corlatti et al. (2019). It is important to stress that, although this dichotomic classification is unlikely to fully capture interindividual variability (e.g., owing to variation in home‐range size and behavioral patterns displayed by males, *cf*. Corlatti et al., 2012; Corlatti et al., 2019), the occurrence of territorial and nonterritorial behavior has been long recognized in chamois (*cf*. Krämer, 1969) and in several other ungulates (Maher & Lott, 1995; Owen‐Smith, 1977). Furthermore, our classification proved effective at capturing major variations in several chamois traits; for example, T and NT showed significant differences in altitudinal range (Corlatti et al., 2020; Corlatti, Bassano, et al., 2013), stress physiology (Corlatti, 2018; Corlatti et al., 2012, 2014), testosterone levels (Corlatti et al., 2012, 2019), parasite burden (Corlatti et al., 2012, 2019), foraging behavior (Corlatti & Bassano, 2014; Corlatti, Bassano, et al., 2013), rutting behavior (Corlatti, Caroli, et al., 2013), mating success (Corlatti et al., 2012), and reproductive success (Corlatti, Bassano, et al., 2015).

### 2.2 Data collection

Between February 2010 and December 2013, we collected 1 location (fix)/7 hr for each animal. Only high‐quality GPS locations were used. Overall, we collected 33,367 GPS fixes on 12 territorial and 16 nonterritorial males. Not all individuals were sampled over the entire period: Each chamois was monitored for a median of 26 months (min. 3, max. 37 months), for a median of 1,308 fixes/individual (interquartile range [IR]: 758, 1531) and a median frequency of 52 fixes/month/individual (IR: 32, 61) and 2 fix/day/individual (IR: 1, 2).

The QGIS software (QGIS Development Team, 2020) was used to extract the geomorphological variables from a 10 × 10 m digital elevation model (Tarquini et al., 2007, 2012) covering the entire study area. Specifically, we extracted information about altitude (in m), slope (in °), and aspect (southness). Southness was calculated as *‐cos(α),* where *α* is the aspect in radians of the 10 × 10 m square cell and varies from −1 (complete exposure to north) to +1 (complete exposure to south). We acknowledge that part of the variance in habitat selection may be explained by among‐year variations in climatic conditions. Indeed, climatic conditions alone would likely not suffice, and a spatially explicit account of different conditions experienced by animals in terms of, for example, pasture quality, local temperature conditions, and snow accumulation would be desirable. For this reason, in addition to the orographic variables, we included NDVI values as a proxy of vegetation productivity and quality (e.g., Pettorelli et al., 2007). NDVI data were obtained using the package *MODIStsp* (Busetto & Ranghetti, 2016) for R *v*. 4.0.4 (R Development Core Team, 2020) in RStudio *v*. 1.3.1056 (RStudio Team, 2020).

### 2.3 Statistical analysis

GPS coordinates of all chamois locations were imported in QGIS, and the corresponding value of the environmental variables was assigned to each fix. To define habitat availability (see paragraph 2.3), for each GPS fix, we generated a set of 10× points, randomly located within an area defined by a 95% kernel density estimation (Silverman, 1986) encompassing all GPS fixes, and we assigned the same set of variable values as we did for the real locations. GPS locations and random points were handled using the R packages *sp* (Pebesma & Bivand, 2005), *rgdal* (Bivand et al., 2019), *rgeos* (Bivand & Rundel, 2019), *raster* (Hijmans, 2019), and *adehabitatHR* (Calenge, 2006).

Habitat selection was analyzed by generating resource selection probability functions following a “use versus availability” approach (Boyce et al., 2002). In particular, to test the effect of the orographic variables and NDVI on the probability of a positive classification, we compared the variables measured at real chamois locations (1) with those measured at random locations (0) fitting generalized linear mixed models assuming a binomial conditional distribution, using the package *lme4* (Bates et al., 2015). To investigate differences in patterns of resource selection between territorial and nonterritorial males, we included as predictors in our models the two‐way interactions between each explanatory variable (altitude, slope, southness, and NDVI) and a factor variable with two levels describing the mating behavior (T = “territorial”; NT = “nonterritorial”). As animals would seldom select extreme values of altitude, slope, and southness, to capture potential curvilinear relationships with chamois occurrence, quadratic terms for the orographic variables (and their interaction with mating behavior) were included in the linear predictor, and quadratic models were compared with simpler linear models based on their AIC values (Burnham & Anderson, 2002). Individual ID was set as a random intercept in all models to account for correlation that stemmed from repeated sampling of the same individuals.

As opposed to highly dimorphic mountain ungulates (e.g., bighorn sheep *Ovis canadensis* or Alpine ibex *Capra ibex*), where the adoption of ARTs largely depends on age, body mass, and horn size (Pelletier & Festa‐Bianchet, 2006; Willisch et al., 2012), territorial and nonterritorial males do not show significant differences in terms of age or morphological features (*cf*. Corlatti et al., 2012; Corlatti, et al., 2019). Lack of major morphological differences is further supported by the occurrence of compensatory growth in body mass and horn size in chamois (Corlatti, Gugiatti, et al., 2015; Rughetti & Festa‐Bianchet, 2010), which tends to reduce individual differences among adult male chamois. Biometric information was thus not included in the models. Individuals consistently adopted the same mating tactic over several years and had similar home‐range size in different years (Cotza et al., own data); thus, we assumed consistency in the individual ART.

We fitted one model for each of the four seasons, defined according to the period of the year and the biology of the species: spring (April–June, beginning of territorial behavior and birth season, beginning of snow melting), summer (July–September, warmest period), autumn (October–December, mating season, first snowfall), and winter (January–March, coldest period) (*cf*. Viana et al., 2018, for another mountain ungulate, the Iberian ibex *Capra pyrenaica*). To validate the final models, we calculated Tjur's *R* ^2^ (Tjur, 2009) and tested the performance of each model by the percentage of correct classifications of original cases and receiver operating characteristic (ROC) curve analysis, using the R packages *performance* (Lüdecke et al., 2019) and *pROC* (Robin et al., 2011).

## 3 RESULTS

**FIGURE 2:**
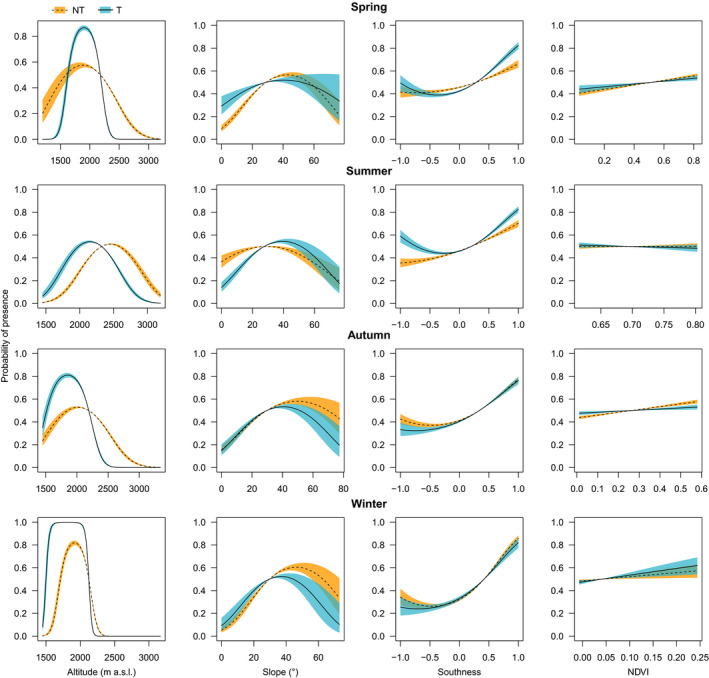
Marginal effects of the models fitting the two‐way interaction between mating behavior (T = “territorial,” in blue; NT = “nonterritorial,” in orange) and altitude (in m a.s.l.), slope (in °), southness (−1/+1), and normalized difference vegetation index (NDVI; 0/+1) to explore seasonal habitat selection in male chamois in Gran Paradiso National Park between 2010 and 2013

Table 1 reports the estimates of the coefficients of the four fitted models. Figure 2 shows the effects of the interaction between environmental variables and mating behavior. All models returned high values for the ROC curve, had high percentages of correct classification, and explained much of the variation in the response variables, with weak individual effect (Table 1). For all response variables, models without quadratic effects in the linear predictor were always less competitive (i.e., they had lower AIC values) than models with quadratic effects (Table 1).

**TABLE 1:** Results of generalized mixed effect models for habitat selection by male chamois in Gran Paradiso National Park between 2010 and 2013. The table reports the beta‐coefficients (±*SE*) for every season and for the rutting period. T = territorial males, *β* = model coefficients, *SE* = standard error, “***”: *p* <.001, “**”: *p* <.01, “*”: *p* <.05, and “.”: *p* <.1. On bottom, the table reports the value of the ROC curve, the explained variance (marginal and conditional *R* ^2^), the percentage of correct classification, Akaike's information criterion (AIC), and AIC of the same model without the quadratic terms (AIC_linear_)

Variable	Spring			Summer			Autumn			Winter		
	*β* (±*SE*)	*p*		*β* (±*SE*)	*p*		*β* (±*SE*)	*p*		*β* (±*SE*)	*p*	
(Intercept)	−2.49 (±0.058)	<.001	***	−1.85 (±0.241)	<.001	***	−2.77 (±0.050)	<.001	***	−7.69 (±0.158)	<.001	***
Altitude	−1.36 (±0.042)	<.001	***	0.35 (±0.024)	<.001	***	−0.95 (±0.031)	<.001	***	−11.74 (±0.291)	<.001	***
Altitude^2	−0.81 (±0.032)	<.001	***	−0.66 (±0.026)	<.001	***	−0.72 (±0.026)	<.001	***	−5.91 (±0.150)	<.001	***
Slope	0.55 (±0.028)	<.001	***	0.060 (±0.02)	.001	**	0.50 (±0.025)	<.001	***	0.68 (±0.035)	<.001	***
Slope^2	−0.23 (±0.018)	<.001	***	−0.11 (±0.016)	<.001	***	−0.13 (±0.015)	<.001	***	−0.16 (±0.020)	<.001	***
Southness	0.37 (±0.019)	<.001	***	0.46 (±0.018)	<.001	***	0.65 (±0.018)	<.001	***	0.97 (±0.027)	<.001	***
Southness^2	0.24 (±0.033)	<.001	***	0.19 (±0.029)	<.001	***	0.48 (±0.032)	<.001	***	0.53 (±0.045)	<.001	***
NDVI	0.16 (±0.066)	.015	*	−0.18 (±0.343)	.592		0.19 (±0.070)	.007	**	0.65 (±0.327)	.046	*
Mating(T)	−3.70 (±0.153)	<.001	***	−0.79 (±0.365)	.030	*	−2.18 (±0.113)	<.001	***	−12.25 (±0.557)	<.001	***
Altitude*mating(T)	−7.58 (±0.260)	<.001	***	−1.41 (±0.050)	<.001	***	−5.47 (±0.181)	<.001	***	−20.04 (±0.96)	<.001	***
Altitude^2*mating(T)	−3.48 (±0.131)	<.001	***	−0.19 (±0.044)	<.001	***	−2.20 (±0.090)	<.001	***	−7.49 (±0.417)	<.001	***
Slope*mating(T)	−0.39 (±0.041)	<.001	***	0.45 (±0.039)	<.001	***	0.02 (±0.040)	.583		−0.30 (±0.052)	<.001	***
Slope^2*mating(T)	0.11 (±0.027)	<.001	***	−0.08 (±0.027)	.003	**	−0.09 (±0.026)	<.001	***	−0.11 (±0.035)	.001	***
Southness*mating(T)	0.22 (±0.030)	<.001	***	0.00 (±0.029)	.940		0.12 (±0.031)	<.001	***	0.02 (±0.044)	.703	
Southness^2*mating(T)	0.26 (±0.054)	<.001	***	0.39 (±0.049)	<.001	***	−0.35 (±0.050)	<.001	***	−0.08 (±0.076)	.291	
NDVI*mating(T)	0.00 (±0.103)	.965		0.11 (±0.514)	.838		−0.14 (±0.105)	.194		0.03 (±0.504)	.946	
Area under ROC curve	86.9			74.3			87.3			95.0		
Conditional *R* ^2^	94.5			34.3			90.2			94.5		
Marginal *R* ^2^	94.5			34.2			90.2			90.5		
Percentage of correct classification	90.8			90.9			90.9			92.4		
AIC	37,707.7			41,774.0			48,752.6			29,435.5		
AIC_linear_	42,453.2			44,065.9			53,155.2			39,197.6		

### 3.1 Altitude

The interaction between mating behavior and altitude (quadratic effect) was always significant, suggesting different use of elevation between the two alternative reproductive tactics throughout the year. In particular, territorial males generally selected lower altitudes than nonterritorial ones (Table 1). The models showed that in spring, both territorial and nonterritorial males were more likely to be found at altitudes between 1,800 and 2,000 m, but territorial males showed a narrower preference for the areas around 1,900 m whereas nonterritorial males selected a wider altitudinal range (Figure 2). In summer, both male types moved upward but the model suggested a lower overlap than in spring, with territorial males selecting altitudes around 2,200 m and nonterritorial males altitudes around 2,500 m (Figure 2). In autumn, the difference between alternative tactics was slightly less evident than in summer, with territorial males finding an optimum between 1,800 and 1,900 m, while nonterritorials selected the same elevations observed in spring, between 2,000 and 2,200 m (Figure 2). In winter, both tactics tended to select lower altitudes, although nonterritorials showed an optimum of preference at 2,000 m while territorial males tended to select even lower altitudes (Figure 2).

### 3.2 Slope

In general, both territorial and nonterritorial males showed preference for slopes between 30 and 50° in spring and in summer and for steeper slopes between 40 and 60° in autumn and winter. Significant differences in the effect of slope on a positive classification of points between the two reproductive tactics were evident throughout the year (Table 1). In spring, although the use of slope in two alternative tactics showed a large overlap, the gentler slopes were relatively more selected by territorial males (Figure 2). In summer, territorial male chamois selected steeper slopes, with an optimum at about 45°, while nonterritorial males showed a preference for areas at approximately 30° (Figure 2). In autumn, the two tactics showed large overlap in the selected slopes, but nonterritorial males increased their preference for steeper areas (between 40 and 60°), as compared to summer (Figure 2). Similarly, in winter, we observed a significant difference between territorial and nonterritorial chamois: the former selected areas around 35° and the latter positively selected areas between 40 and 60° (Figure 2).

### 3.3 Aspect

The southern exposures were generally always preferred by both male types in all seasons (Table 1). The coefficients of the models show greater selection of southern slopes by territorial males in spring and in summer, indicating that in these periods territorial males are (relatively) more likely to be found on southern‐exposed aspects than nonterritorial ones (Figure 2). In autumn and in winter, differences between the two male types were milder (Figure 2). However, while in winter no significant difference was detected (Table 1) and both ARTs tended to avoid the north‐facing exposures, in autumn this avoidance was greater in territorial males (Figure 2), leading to significant differences between ARTs (Table 1).

### 3.4 NDVI

In general, NDVI values were positively associated with chamois presence in spring, autumn, and winter, indicating a preference for “greener” areas in those seasons. This effect was particularly evident in winter, as suggested by a greater effect size (Table 1). Notably, however, we did not observe any significant difference between territorials and nonterritorial males in NDVI selection in any season.

## 4 DISCUSSION

Males adopting alternative reproductive tactics in chamois profoundly differed in their habitat selection not only during the rutting season, but throughout the year. Compared to nonterritorial males, territorial males used lower elevations especially in summer and autumn, used steeper areas in summer and preferred southern slopes in spring and summer. ARTs did not show difference in the selection of NDVI throughout the year.

In spring, the strong preference of territorial males toward low‐elevation, southern‐exposed slopes may reflect early occupation of territories (von Hardenberg et al., 2000), possibly suggesting a strong influence of the need to increase mating opportunities on their habitat choice, as these areas may be occupied by females during the rut (Corlatti et al., 2020; Lovari et al., 2006). Conversely, habitat preferences in nonterritorial males in spring appeared looser, especially in terms of elevation, and may reflect the need to exploit foraging opportunities; for example, nonterritorial males may tend to follow the upward shift in vegetation green‐up. This suggestion is supported by available data on females, which show that females and nonterritorial males increase their home‐range size and move to higher elevations from late spring, suggesting a more similar movement pattern between these two categories than between females and territorial males (*cf*. Nesti et al., 2010 for comparison among females, migrant, and resident males, and Corlatti et al., 2020). These results are in line with previous studies on other species of mountain ungulates, which undertake seasonal migrations as adaptation to exploit temporal differences in vegetation phenology (Albon & Langvatn, 1992; Bischof et al., 2012; Festa‐Bianchet, 1988; Mysterud et al., 2001) and in response to changes in snow cover, temperature, and solar radiation (Aublet et al., 2009; Richard et al., 2014).

Similarly, also over the summer, territorial males tended to select areas at lower elevation, with steeper and southern‐exposed slopes, compared to nonterritorial ones, which may have exploited the upward shift in vegetation phenology (Albon & Langvatn, 1992; Bischof et al., 2012; Mysterud et al., 2001). We may hypothesize that these areas were positively selected by territorials because they will be snow‐free territories during the rutting season, thus attractive to females (Corlatti et al., 2020; Lovari et al., 2006). Indeed, mating opportunities are expected to still play a major role in habitat selection of territorial chamois in this time of the year, as dominant males should defend their territories until autumn (von Hardenberg et al., 2000), whereas habitat choice in nonterritorial males should be mainly driven by foraging opportunities. This hypothesis is supported by the fact that chamois female spatial behavior, which is less constrained than male spatial behavior by reproductive needs, is closer to the nonterritorial than to the territorial spatial behavior. If so, territorial behavior may hamper energy acquisition, because crude protein content in vegetation typically increases with altitude in summer (Albon & Langvatn, 1992; Van Soest, 1994). Therefore, habitat choice in territorial males likely reflects a complex combination of mating and foraging opportunities and costs. Furthermore, these trade‐offs may change depending on individual heterogeneity. Some territorial males, for example, may stay at lower elevations throughout the summer, in order not to lose their territory, while a few others may afford to temporarily abandon their territory and return to it before the rut (own data), thus exploiting the higher forage quality found at higher elevations.

In autumn, mating opportunities are expected to play a greater role in habitat selection than foraging opportunities in both male types: In the November rut, males tend to lower their food intake in favor of mating effort (Willisch & Ingold, 2007), although the occurrence of hypophagia is more evident in territorial than in nonterritorial males (Corlatti & Bassano, 2014). All in all, we may expect a relatively high degree of overlap in the home ranges of males, thus nonsignificant differences in habitat selection, owing to the frequent male–male interactions (Corlatti et al., 2012). However, while both male types positively selected southern‐exposed steep areas, there was a stronger selection for low elevations in territorial than in nonterritorial males. A possible explanation for this pattern is that the dominance expressed by territorial males may have forced nonterritorial males to occupy areas outside the territories (e.g., above the territories). Nonetheless, we should also point out that the difference in elevation between male types could also reflect variation in mating opportunities over different years, irrespective of male–male contests. In years with little snow cover, nonterritorial males may remain at higher elevations, where the majority of females are, while territorial males would be forced to remain at lower elevations, in their territories (Corlatti et al., 2020; Lovari et al., 2006).

Mating opportunities are unlikely to play a major role in habitat selection during winter, as territoriality is not expressed in this period. Rather, winter climate may force both male types to occupy sites with greater foraging opportunities, that is, steep, southern‐exposed slopes at low elevations, likely snow‐free. Such areas would further favor thermoregulation; Brivio et al. (2016), for example, found that chamois activity was positively related to radiation during the winter months and suggested that chamois could benefit from the absorption of solar radiation in winter. A similar behavior was observed for another mountain ungulate sharing the habitat with chamois, the Alpine ibex (Signer et al., 2011). Nonetheless, nonterritorial males positively selected for steeper slopes at seemingly higher elevations, compared to territorial males. We may hypothesize that after the great energy expenditures that occur during the rutting period, territorial males may temporarily lose their dominance over nonterritorial ones, which may take over the best wintering sites, that is, those that granted little snow accumulation. Alternatively, we may also hypothesize that some territorial males, after the rut, may move to even lower elevations, where they do not need to select for steeper slopes, as those areas will be already snow‐free.

Notably, our results suggest that all males, irrespective of the mating tactic, selected “greener” areas (i.e., with higher values of NDVI) in spring, autumn, and especially in winter. The lack of difference between territorial and nonterritorial males in the selection of vegetation productivity seemingly contradicts the hypothesized differences in terms of foraging opportunities between ARTs, especially in spring and summer. The use of NDVI as an index of vegetation productivity associated with ARTs, however, needs to be treated cautiously, as the direct use of NDVI values as a proxy of bromatological variables (e.g., crude protein, neutral and acid detergent fiber, and aboveground biomass, indicators of plant quality and nutrient contents) is weakly supported in our study area (Ranghetti et al., 2016). Similar values of “greenness” may thus entail different quality of pastures, and data on the forage energetic value associated with the areas used by the two male tactics are needed to further investigate ART‐specific foraging opportunities. A previous study suggested no major differences in crude protein content in the diet of territorial and nonterritorial males throughout the year (Corlatti, Bassano, et al., 2013). In late spring and summer, this lack of difference in ART‐specific dietary quality may owe to greater amount of time spent foraging by territorial males (*cf*. Corlatti, Bassano, et al., 2013). Namely, territorial males may inhabit lower‐food‐quality habitats in spring and summer, compared to nonterritorial males (*cf*. Albon & Langvatn, 1992, Van Soest, 1994), but still increase their dietary quality by spending more time being more selective in their food choice (*cf*. Corlatti & Bassano, 2014). Whether this hypothesized selective behavior may entail greater energetic costs for territorial males still needs to be clarified: In summer, for example, warmer temperatures at low elevations could increase heat stress (Parker, 1988, for black‐tailed deer *Odocoileus hemionus columbianus*; Grignolio et al., 2004, for Alpine ibex; van Beest et al., 2011, for moose *Alces alces*) and possibly limit the energy gain by constraining foraging time (Aublet et al., 2009; Mason et al., 2014).

Information on habitat selection of alternative reproductive tactics is limited (*cf*. Taborsky et al., 2008). To our knowledge, this study represents the first attempt to investigate the year‐round variation in habitat choice of a large mammal displaying ARTs. Different patterns of habitat selection may reflect different trade‐offs between opportunity for survival and reproduction in ARTs. Our results suggest that territorial behavior may possibly force males into warmer, lower‐food‐quality habitats, during late spring and summer, whereas nonterritorial males may have the opportunity to follow and exploit vegetation phenology and more favorable temperatures: Trade‐offs may thus develop between the optimization of energy balances before the rut and the increase of mating opportunities during the rut in ARTs (*cf*. Corlatti, Caroli, et al., 2013). Finally, while our study suggests that individual year‐round variation in habitat selection may be dictated by the occurrence of ARTs, the origin of alternative male mating behaviors itself may largely depend on the spatial distribution of females (Corlatti et al., 2020; Lovari et al., 2006). In turn, this suggests that linking alternative male reproductive tactics and male habitat selection is a complex issue that entails the indirect effect of female spatial behavior (*cf*. Emlen & Oring, 1977).

## CONFLICT OF INTEREST

We have no competing interests.

## AUTHOR CONTRIBUTION

**Luca Corlatti:** Conceptualization (lead); Data curation (lead); Formal analysis (supporting); Investigation (lead); Methodology (equal); Supervision (lead); Writing‐original draft (lead); Writing‐review & editing (lead). **Antonella Cotza:** Conceptualization (supporting); Writing‐original draft (supporting); Writing‐review & editing (supporting). **Luca Nelli:** Conceptualization (supporting); Data curation (supporting); Formal analysis (lead); Methodology (equal); Visualization (lead); Writing‐original draft (supporting); Writing‐review & editing (supporting).

## Data Availability

Data used in this analysis are available at Dryad Digital Repository https://doi.org/10.5061/dryad.cnp5hqc4g
